# Supplementary material for: Self-assembly of 1T/1H superlattices in transition metal dichalcogenides
Source: Nat Commun. 2024 Dec 4;15:10584. doi: 10.1038/s41467-024-54948-x (PMC11618666; doi:10.1038/s41467-024-54948-x)
Supplement: Supplementary file 1 — Supplementary Information [file 41467_2024_54948_MOESM1_ESM.pdf]

Supplementary Information for

Self-assembly of 1T/1H superlattices in transition metal dichalcogenides

Chaojie Luo[1,2,3#], Guohua Cao[1,2,3#], Beilin Wang[1,2,3#], Lili Jiang[1,2,3], Hengyi Zhao[1,2,3], Tongrui Li[4], Xiaolin Tai[5], Zhiyong Lin[1,2,3], Yue Lin[1], Zhe Sun[4], Ping Cui[1,2,3]*, Hui Zhang[1,2,3]*, Zhenyu Zhang[1,2,3], Changgan Zeng[1,2,3]*

[1]*International Center for Quantum Design of Functional Materials (ICQD), Hefei National Research Center for Physical Sciences at the Microscale, University of Science and Technology of China, Hefei, Anhui 230026, China*

[2]*CAS Key Laboratory of Strongly-Coupled Quantum Matter Physics, and Department of Physics, University of Science and Technology of China, Hefei, Anhui 230026, China*

[3]*Hefei National Laboratory, University of Science and Technology of China, Hefei 230088, China*

[4]*National Synchrotron Radiation Laboratory, University of Science and Technology of China, Hefei 230029, China*

[5]*Department of Chemistry, University of Science and Technology of China, Hefei 230029, China*

# These authors contributed equally.

* Correspondence and requests for materials should be addressed to C. Z. (cgzeng@ustc.edu.cn), H. Z. (huiz@ustc.edu.cn) and P. C. (cuipg@ustc.edu.cn).

Supplementary Note 1: Structural analysis of the $NbSe_{2-x}Te_x$ single crystals (x < 0.5, x > 1.1)

To determine the exact structures of $NbSe_{2-x}Te_x$ single crystals when $x < 0.5$ and $x > 1.1$, we performed STM and STEM characterizations. Supplementary Figure 2a shows the STEM image of a $NbSe_{2-x}Te_x$ single crystal at $x = 0.28$. The 1H layers with trigonal prismatic coordination are stacked into the bulk following AA' stacking sequences, where adjacent 1H layers rotate 60° around a central Nb atom, known as the 2H type[1,2]. Supplementary Figures 3a and 3b display atomic-resolution STM images of the 2H type with different Te concentrations. The random bright spots in topography images,

associated with the substituted Te atoms, increase with the elevation of Te concentration (Supplementary Fig. 3a, b).

Supplementary Figure 2b presents the STEM image of a $NbSe_{2-x}Te_x$ single crystal at $x = 1.48$. The 1T layers with octahedral coordination are stacked into the bulk following AA stacking sequences, known as the 1T type[1,2]. Supplementary Figures 3c-f show atomic-resolution STM images of the 1T type with different Te concentrations and their associated Fast Fourier transform (FFT) images. The 1T type with different Te concentrations exhibit short range 1×3 reconstruction[3] (Supplementary Fig. 3c, e), and the 1×3 reconstruction peak can also be observed in the FFT images of topography images along three directions (Supplementary Fig. 3d, f).

In conclusion, $NbSe_{2-x}Te_x$ single crystals exhibit a 2H type when $x < 0.5$ and transition to distorted 1T type (with 1×3 reconstruction) when $x > 1.1$.

Supplementary Note 2: Reconstruction of the 1T layer in 1T/1H superlattice

To elucidate whether the 1T layers in the 1T/1H superlattice undergo reconstruction, we performed atomic-resolution STM characterization. Supplementary Fig. 7a illustrates various terminations within the 1T/1H superlattice. The termination on the left half of the step shows short range reconstruction compared to that on the lower left step. In the zoomed-in atomic-resolution STM image of the left termination (Supplementary Fig. 7b), the star-of-David reconstruction is distinctly visible, and the associated FFT image further confirms the presence of the star-of-David reconstruction[4]. This observation aligns with the star-of-David reconstruction identified in the 1T layer of the TaS_2 1T/1H superlattice[5,6].

In the zoomed-in atomic-resolution STM image of the termination on the lower right step (Supplementary Fig. 7d), random bright spots can be observed, similar to those in the 1H layer of the 2H-$NbSe_{2-x}Te_x$, attributed to substituted Te atoms. The corresponding FFT image does not reveal any ordered reconstruction. Thus, through atomic-resolution STM images, we can distinguish between the terminations of the 1T and 1H layers, with the termination of the 1T layer exhibiting the star-of-David reconstruction.

Supplementary Note 3: Charge transfer in the 1T/1H superlattice

Here, the charge transfer between the 1T and 1H layers is considered to exist in the 1T/1H superlattice, leading to electrostatic binding among the 1T and 1H layers and eventually contributing to the self-assembly into the 1T/1H superlattice. The work functions of the 1T and 1H layers are 5.4 and 5.83 eV, respectively (see Fig. 2d), which indicates that the 1T layers are likely to transfer charge to the 1H layers, as illustrated in Supplementary Fig. 9a. In order to evaluate the charge transfer in the 1T/1H superlattice, we calculated the differential charge density and corresponding *ab*-plane-averaged

charge density difference of the 1T/1H superlattice in $NbSe_{2-x}Te_x$ with x = 0.72. As shown in Supplementary Fig. 9b, the charge transfer of approximately 0.5 e per unit cell from the 1T to 1H layers is obtained.

Supplementary Note 5: Effects of reconstructions on electron distribution in STEM images of 1T/1H superlattices

In Fig. 2a, the electron distribution in the 1T layer displays a dumbbell-like shape. We speculate that this distinct distribution results from reconstruction-induced lateral local deviations of some Nb atoms along the [110] direction. As shown in Supplementary Fig. S7, we identified notable complex reconstructions within the 1T layers and recognized short-range star-of-David reconstructions, whereas no such reconstruction exists in the 1H layer. Figure S12a confirms the presence of these reconstructions at room temperature in the 1T layer. Since it is challenging to describe such complex reconstructions, our model only considers the star-of-David reconstructions. Figures S12b and S12c depict an atomic model of a $NbSe_{2-x}Te_x$ 1T layer with the star-of-David reconstruction, clearly exhibiting lateral local deviations of the Nb atoms. Based on this evidence and analysis, these reconstructions in the 1T layer are responsible for the observed dumbbell-like distribution of the Nb atomic column in the $NbSe_{2-x}Te_x$ 1T/1H superlattices.

In Fig. 4, for the $Nb_{1-x}V_xSe_2$, the metal atoms at the 1H and 1H' sites appear brighter than those at the 1T and 1T' sites. Additionally, this phenomenon is not as clearly observed in the $Nb_{1-x}Ti_xSe_2$. We performed STEM-EDS mapping on the $Nb_{0.86}V_{0.14}Se_2$ 1T/1H superlattice, as shown in Fig. S13. The results indicate that V atoms exhibit the same spatial distribution and similar intensity in both the 1T and 1H layers, suggesting that their doping concentrations are essentially identical. We propose that this brightness difference is also linked to the reconstruction in the 1T layer. As shown in Fig. S14, both the $Nb_{1-x}V_xSe_2$ and $Nb_{1-x}Ti_xSe_2$ systems exhibit a star-of-David reconstruction in the 1T layer at room temperature. However, this reconstruction is more ordered in the $Nb_{1-x}V_xSe_2$ than in the $Nb_{1-x}Ti_xSe_2$. This may result in the metal atoms at the 1H and 1H' sites to appear brighter than those at the 1T and 1T' sites in the $Nb_{1-x}V_xSe_2$.

Supplementary Note 6: Effects of reconstructions on the formation energy of 1T/1H superlattice

From our DFT calculations, it is indeed observed that the distorted 1T type exhibits slightly lower energies by 1.7 and 3.1 meV per formula unit for x = 0.72 and x = 0.83, respectively, compared to the corresponding 1T/1H superlattice. However, it is important to note that, because the computations were too demanding, the star-of-David reconstructions observed in the 1T layers of the 1T/1H superlattice (see Supplementary Fig. S7) were not included in our DFT calculations. To partially assess

the impact of these reconstructions, we calculated the energy gains for a freestanding monolayer 1T phase and a simplified 1T/1H superlattice model, both incorporating star-of-David reconstructions in their 1T layers, especially for $NbSe_{2-x}Te_x$ with $x = 0.77$. In the simplified 1T/1H superlattice model, the 1T layer in each unit cell is shifted by one-third of the lattice constant relative to the 1H layer along the $[1\bar{1}0]$ direction. As illustrated in Fig. S15, the energy gain for the freestanding monolayer 1T phase is 6.3 meV per formula unit, while this value increases to 17.7 meV per formula unit in the simplified 1T/1H superlattice model. These findings suggest that the presence of star-of-David reconstructions can substantially reduce the formation energy of the 1T/1H superlattice, making it significantly lower than that of the distorted 1T type within the range of $0.64 \leq x \leq 0.89$, thereby facilitating the formation of the 1T/1H superlattice.

Supplementary Note 7: Mixed phases in $NbSe_{2-x}Te_x$ single crystals

In Fig. 1a, we confirm that $NbSe_{2-x}Te_x$ single crystals form a 1T/1H superlattice in the range of $0.64 \leq x \leq 0.89$ and transition to a distorted 1T type for $x > 1.1$. Previous research has indicated that NbSeTe ($x = 1$) exists in the 1T type[7]. To better understand the structure at this composition, we conducted STEM experiments on a single crystal with $x = 1.03$. As shown in Fig. S16, the $NbSe_{2-x}Te_x$ single crystal at $x = 1.03$ comprises a mixture of a distorted 1T type (marked by the red regions) and a 1T/1H superlattice (marked by the blue regions). These results suggest that when $x \approx 1$, $NbSe_{2-x}Te_x$ is in a transitional phase between the 1T/1H superlattice and distorted 1T type. Therefore, it is reasonable that the previous work reported NbSeTe as a 1T type structure at $x = 1$, since different growth conditions may lead to structural variations for the transitional phase.

Supplementary Note 8: Superconductivity of $NbSe_{2-x}Te_x$ single crystals

We conducted low-temperature transport experiments to investigate the superconductivity of $NbSe_{2-x}Te_x$ single crystals. As shown in Fig. S17, the $NbSe_{2-x}Te_x$ 1T/1H superlattice exhibits superconductivity, with a transition temperature between 1.6 and 2.5 K, positioned between $NbSe_2$ and $NbTe_2$. The superconducting transition temperatures of the 1T/1H superlattice are nearly identical along different crystal orientations.

Supplementary Note 9: Formation mechanism of the 1T/1H superlattice

$NbSe_2$ and $NbTe_2$ usually stabilize in the 2H type and 1T'' type, respectively. However, when forming $NbSe_{2-x}Te_x$ superlattices, there is a distinct tendency to stabilize in the 1T/1H superlattice rather than in the 2H/1T'' or other superlattice configurations. This preference can be analyzed through lattice mismatch considerations. For a given x in $NbSe_{2-x}Te_x$, the 1H and 1T layers usually exhibit very similar lattice constants. However, the 1T'' layer, which undergoes a 1×3 reconstruction, possesses a significantly larger lattice constant along the reconstruction direction compared to the corresponding reorganized lattice constant of the 1H or 1T layer. For example, the lattice mismatch between the 1H and 1T layers for $NbSe_2$ ($NbTe_2$) is 0.077% (0.38%), while the mismatch between the 1H and 1T'' layers is 1.6% (1.7%). Such larger mismatches between the 1H and 1T'' layers can lead to higher energies when forming superlattices. As a consequence, the $NbSe_{2-x}Te_x$ superlattice tends to stabilize in the 1T/1H superlattice rather than in the structures involving the 1T'' layers. From Fig. 3d, we find that the formation energy of the most stable 2H/1T'' configuration is indeed higher than that of the 1T/1H (6R) configuration.

Supplementary Figures

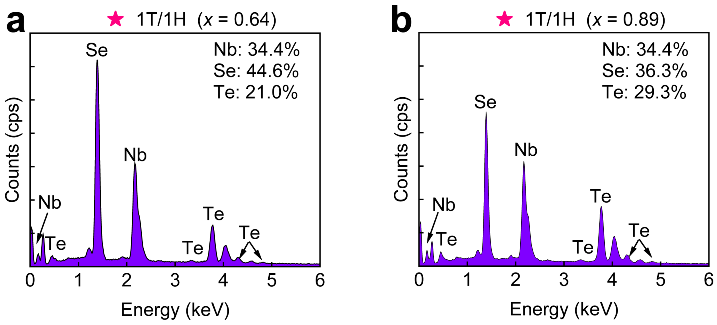

Supplementary Fig. 1 | Stoichiometric ratio of 1T/1H superlattice. a, **b** EDS spectra of 1T/1H superlattice. The stoichiometric ratio of Nb : Se : Te are 1.03 : 1.34 : 0.64 in (**a**) and 1.03 : 1.11 : 0.89 in (**b**).

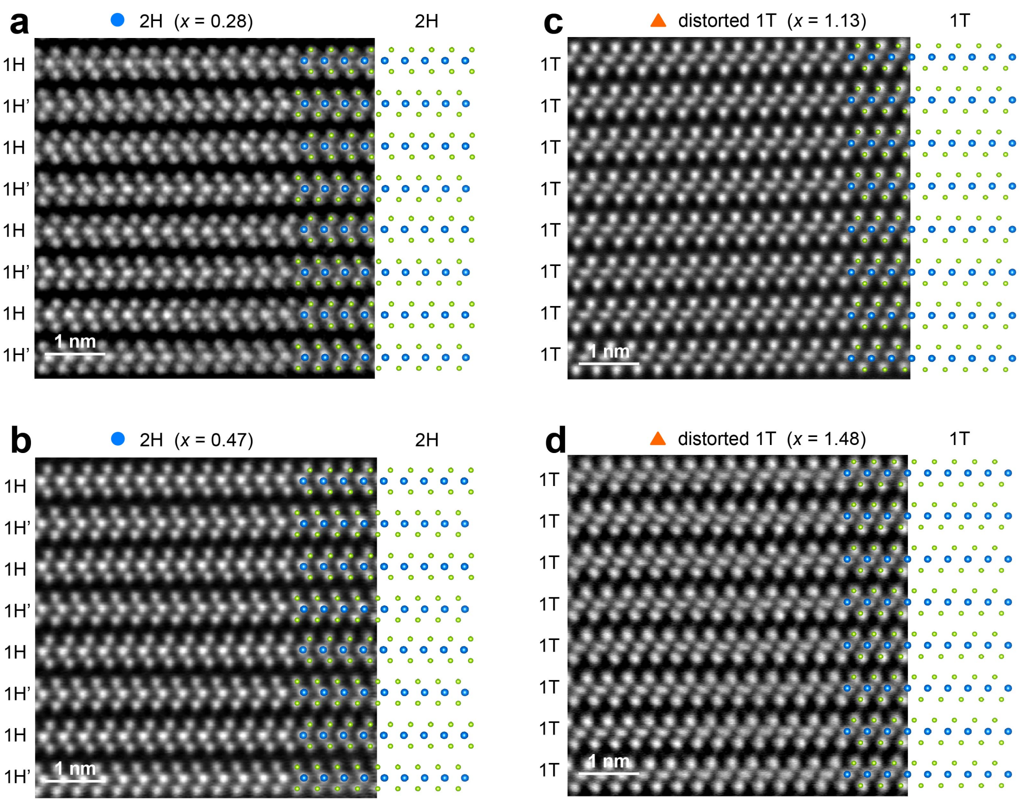

Supplementary Fig. 2 | Atomic-scale structures of 2H and distorted 1T types. a, **b** STEM images of 2H structures with $x = 0.28$ and 0.47. **c, d** STEM images of distorted 1T structures with $x = 1.13$ and 1.48.

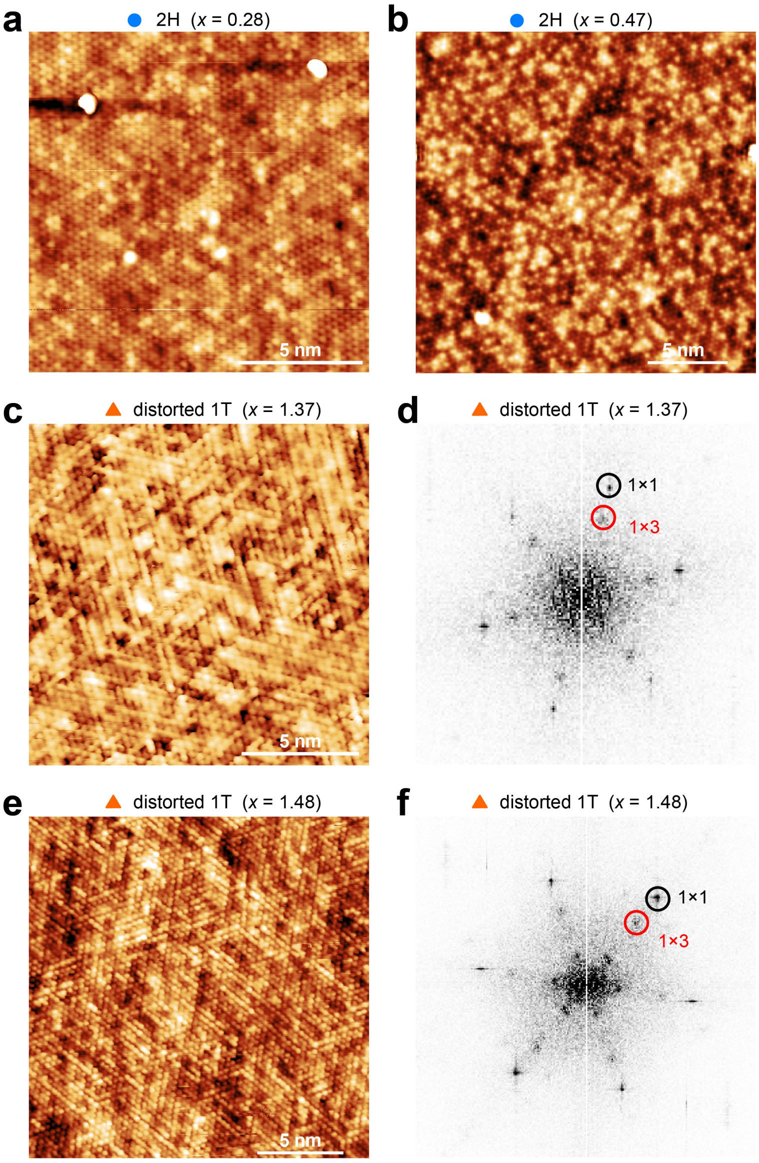

Supplementary Fig. 3 | Atomic-resolution STM images of 2H type and distorted 1T type with 1×3 reconstruction. a, **b** Atomic-resolution STM images of 2H type (x = 0.28, x = 0.47) at T = 77 K. The random bright spots are associated with the substituted Te atoms. Scanning parameters: V_{bias} = 0.5V, I_{set} = 800 pA. **c**, **e** Atomic-resolution STM images of distorted 1T type (x = 1.37, x =1.48) at T = 77 K. Scanning parameters: V_{bias} = 0.1V, I_{set} = 800 pA. **d**, **f** FFT images of STM images. The short range 1×3 reconstruction is clearly shown in FFT images. The black and red circles mark the Bragg and 1×3 reconstruction peak, respectively.

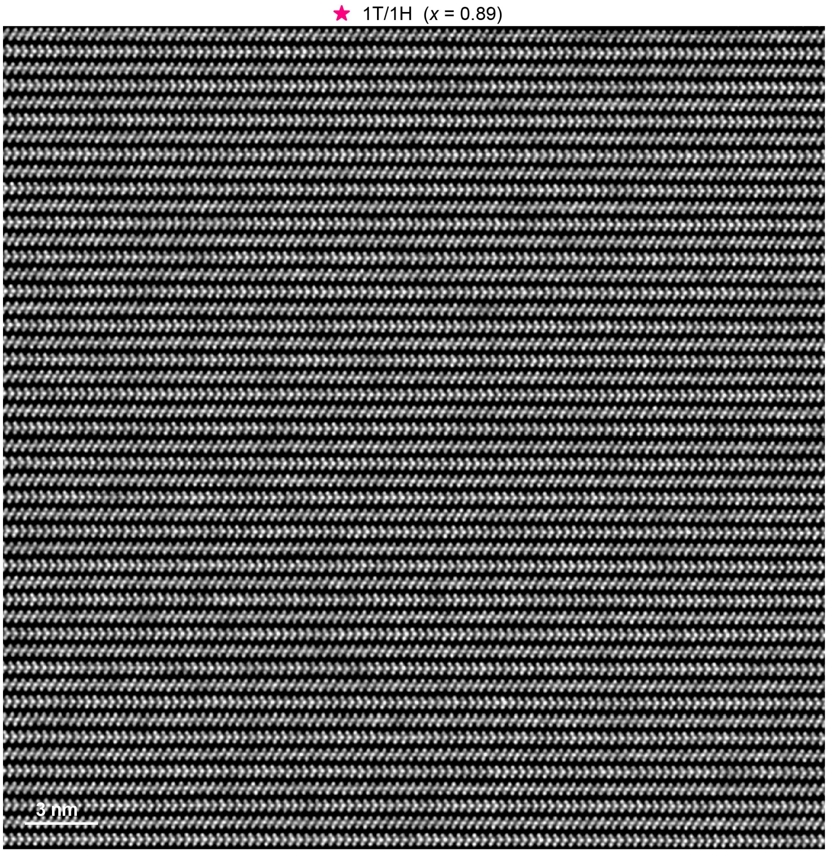

Supplementary Fig. 4 | Homogeneity of 1T/1H superlattice (x = 0.89). Large scale atomic-resolution STEM image of 1T/1H superlattice (x = 0.89).

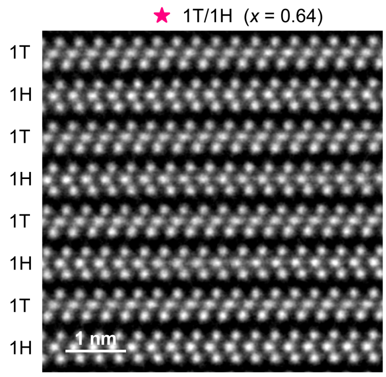

Supplementary Fig. 5 | Atomic-scale structure of 1T/1H superlattice (*x* = 0.64). STEM image of 1T/1H superlattice (*x* = 0.64) crystal viewed along the [110] direction, also showing the alternating arrangement of 1H and 1T layers with 6R structure.

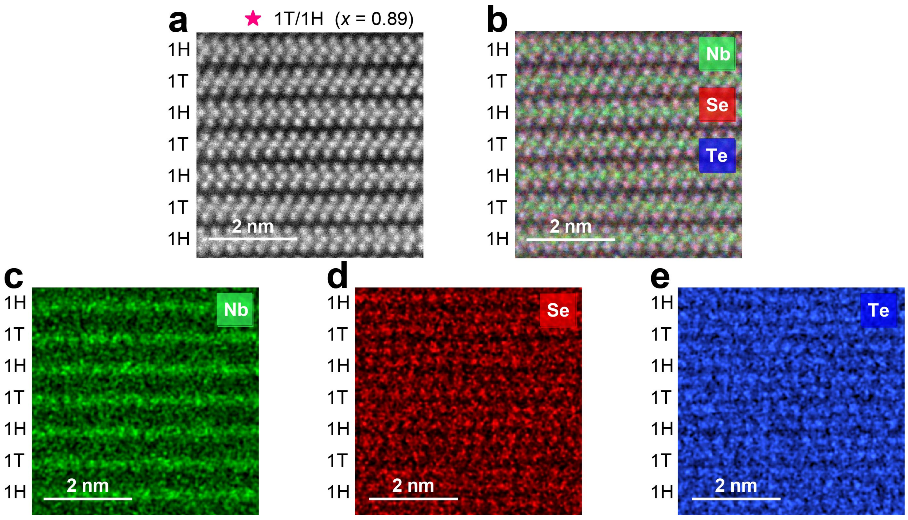

Supplementary Fig. 6 | Atomic-scale structural and chemical analysis of 1T/1H superlattice (x = 0.89). **a** Atomic-resolution STEM image acquired simultaneously with chemical mapping via energy dispersive X-ray spectroscopy imaging. **b** Overlay of the Nb (green), Se (red) and Te (blue) signals. **c** Chemical mapping of Nb. **d** Chemical mapping of Se. **e** Chemical mapping of Te.

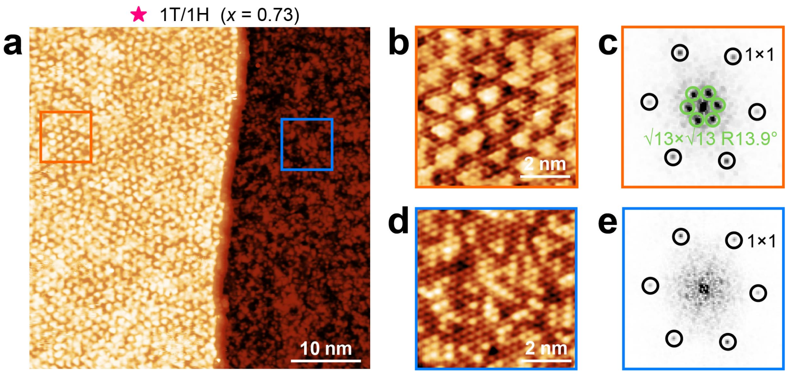

Supplementary Fig. 7 | Different termination surfaces in the 1T/1H superlattice (x = 0.73). a Topography of a cleaved surface showing both 1T and 1H layer terminations at T = 77 K. Scanning parameters: V_{bias} = 1 V, I_{set} = 100 pA. **b, d** Zoomed-in topographies of the 1T (**b**) and 1H (**d**) layer terminations and their corresponding FFT images, (**c**) for 1T layer, (**e**) for 1H layer. Scanning parameters: V_{bias} = 0.1 V, I_{set} = 800 pA. 1T layer termination shows star-of-David reconstruction in (**b**), and the FFT image (**c**) clearly shows the atomic Bragg peak (black), and the star-of-David reconstruction peak (green).

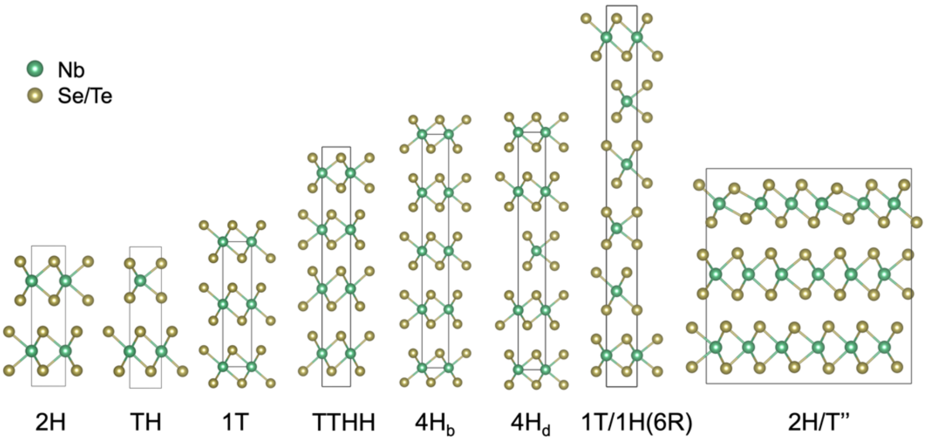

Supplementary Fig. 8 | Eight bulk configurations with different stacking orders of 1H or/and 1T layers for $NbSe_{2-x}Te_x$ with x = 0.83.

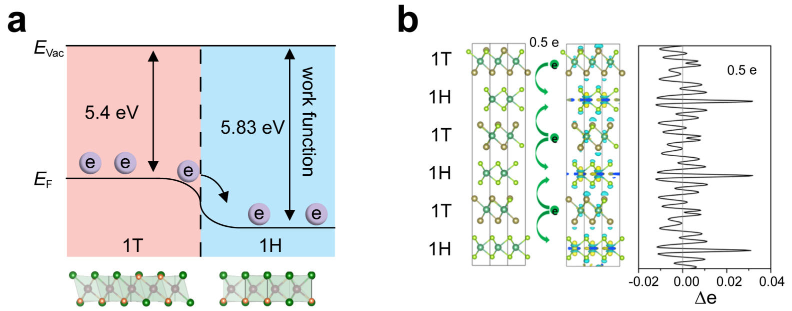

Supplementary Fig. 9 | Charge transfer from 1T layers to 1H layers. a Illustration of the alignment of the Fermi levels (E_F) of the 1T and 1H layers, with the work functions of the 1T (5.4 eV) and 1H (5.83 eV) layers taken from Fig. 2d. The curved arrow denoting the charge transfer direction when forming the 1T/1H superlattice. **b** Crystal structure (left panel), differential charge density (middle panel), and corresponding *ab*-plane-averaged charge density difference (right panel) of the 1T/1H superlattice in $NbSe_{2-x}Te_x$ with $x = 0.72$. In the middle panel, the yellow and blue isosurfaces represent regions of charge accumulation and depletion, respectively.

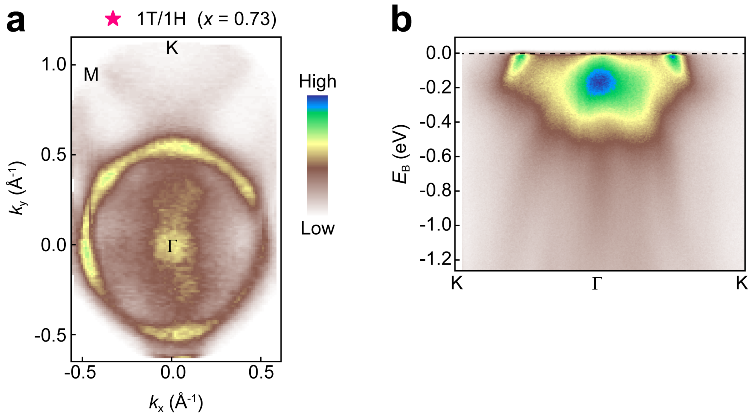

Supplementary Fig. 10 | Electronic structures of the 1T/1H superlattice (*x* = 0.73). a ARPES intensity at the Fermi surfaces of the 1T/1H superlattice (*x* = 0.73) single crystals. **b** Photoemission intensity plots along the Γ−K direction of the 1T/1H superlattice (*x* = 0.73) single crystals. The data were taken using 23 eV photons at *T* = 10 K.

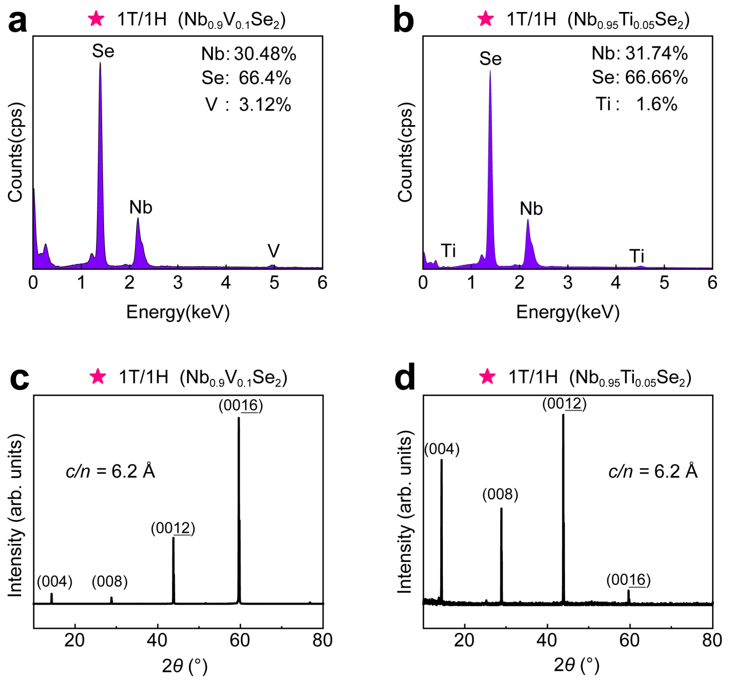

Supplementary Fig. 11 | Stoichiometric ratio and single crystal XRD of $Nb_{0.9}V_{0.1}Se_2$ and $Nb_{0.95}Ti_{0.05}Se_2$. a, b EDS spectra of $Nb_{0.9}V_{0.1}Se_2$ and $Nb_{0.95}Ti_{0.05}Se_2$. The stoichiometric ratio of Nb : V : Se is 0.9 : 0.1 : 2 in (**a**) and Nb : Ti : Se is 0.95 : 0.05 : 2 in (**b**). **c**, **d** XRD characterization of $Nb_{0.9}V_{0.1}Se_2$ and $Nb_{0.95}Ti_{0.05}Se_2$ single crystal. The *c*/*n* of $Nb_{0.9}V_{0.1}Se_2$ and $Nb_{0.95}Ti_{0.05}Se_2$ are both 6.2 Å, which are nearly identical.

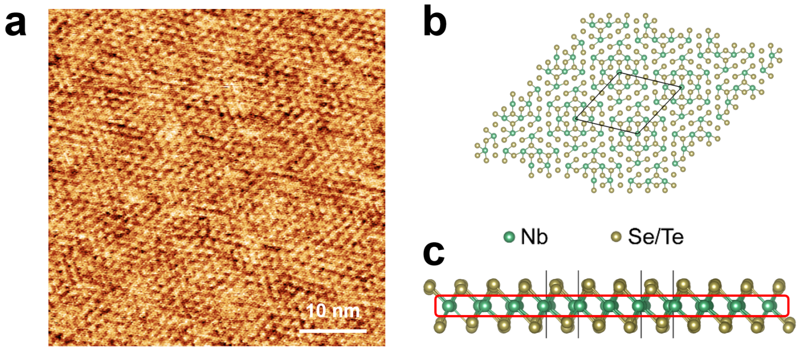

Supplementary Fig. 12 | Reconstruction of the 1T layer in 1T/1H superlattice ($NbSe_{2-x}Te_x$) at room temperature. a Topography of a cleaved surface displaying 1T layer termination in the 1T/1H superlattice at $T = 300$ K. Scanning parameters: $V_{bias} = 0.01$ V, $I_{set} = 2.5$ nA. **b** Top view of the 1T layer featuring the star-of-David reconstruction. **c** Lateral view of the 1T layer with star-of-David reconstruction along the [110] direction.

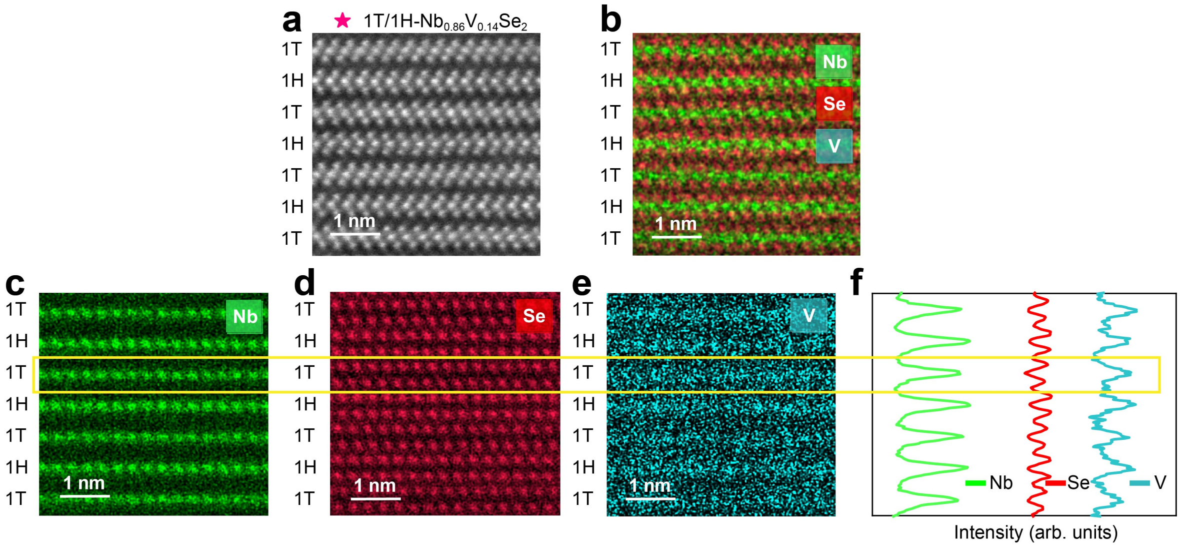

Supplementary Fig. 13 | Atomic-scale structural and chemical analysis of 4H$_b$-Nb$_{0.86}$V$_{0.14}$Se$_2$. a Atomic-resolution STEM image acquired simultaneously with chemical mapping via energy dispersive X-ray spectroscopy imaging. **b** Overlay of the Nb (green), Se (red) and V (cyan) signals. **c** Chemical mapping of Nb. **d** Chemical mapping of Se. **e** Chemical mapping of V. **f** Lateral integration of chemical mapping intensities. The integrated intensity curves are shifted horizontally for clarity.

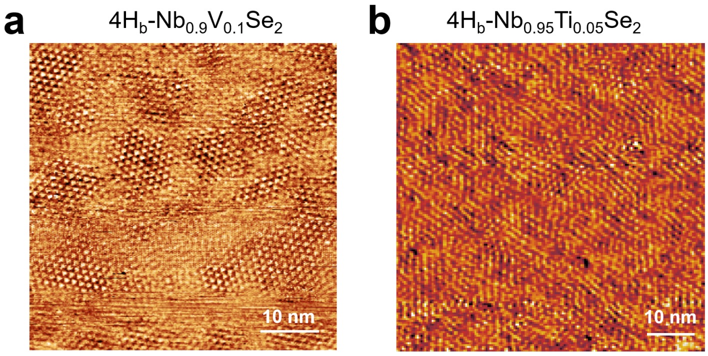

Supplementary Fig. 14 | Reconstruction of the 1T layer in 1T/1H superlattice ($Nb_{1-x}V_xSe_2$, $Nb_{1-x}Ti_xSe_2$) at room temperature. a Topography of a cleaved surface showing 1T layer termination of 4H$_b$-$Nb_{0.9}V_{0.1}Se_2$ at T = 300 K. **b** Topography of a cleaved surface showing 1T layer termination of 4H$_b$-$Nb_{0.95}Ti_{0.05}Se_2$ at T = 300 K. Scanning parameters: V_{bias} = 0.05 V, I_{set} = 2 nA.

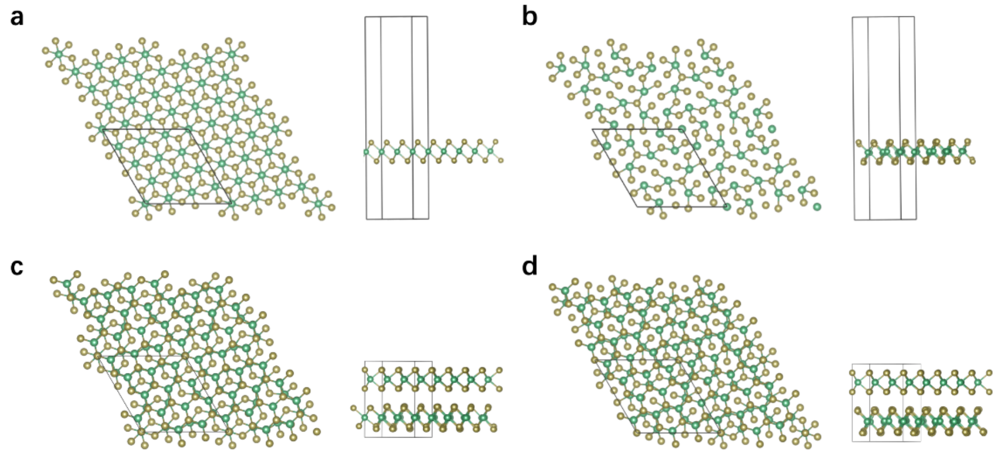

Supplementary Fig. 15 | Crystal structures of two different phases in $NbSe_{2-x}Te_x$ with x = 0.77. Top (left panel) and side (right panel) views of the atomic structures without (**a**) and with (**b**) star-of-David reconstruction for a freestanding monolayer 1T phase. **c**, **d** Same as (**a**, **b**) but for a simplified 1T/1H superlattice model. In the simplified 1T/1H superlattice model, the 1T layer in each unit cell is shifted by one-third of the lattice constant relative to the 1H layer along the $[1\bar{1}0]$ direction.

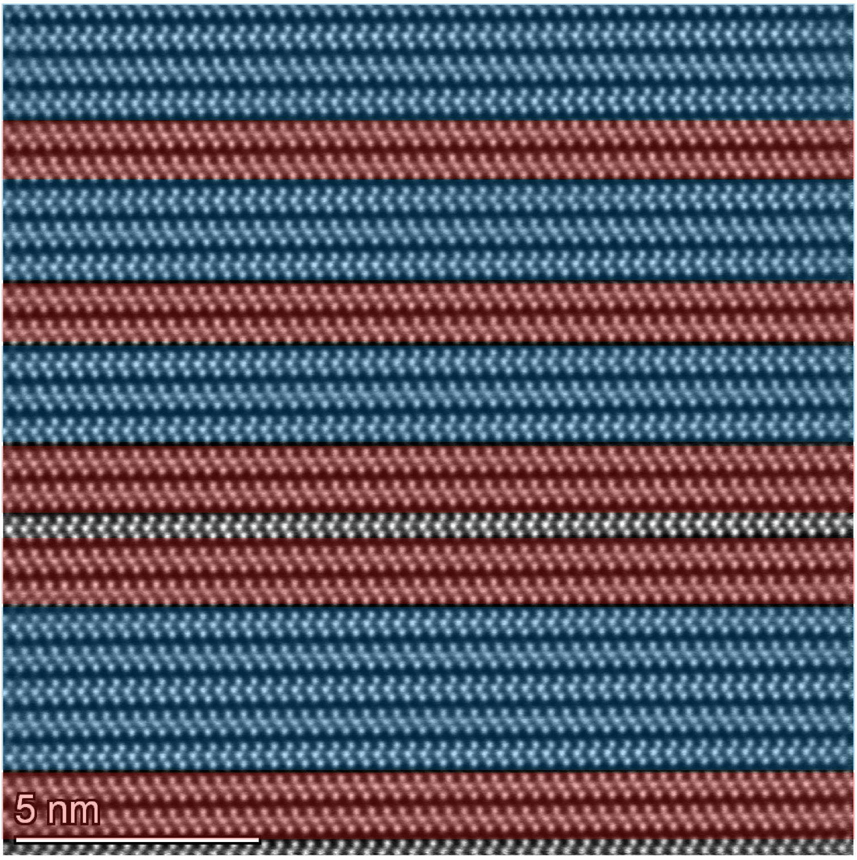

Supplementary Fig. 16 | Atomic-scale structure of $NbSe_{2-x}Te_x$ (x = 1.03). STEM images of $NbSe_{2-x}Te_x$ (x = 1.03). The red regions mark the distorted 1T type and the blue regions mark the 1T/1H superlattice.

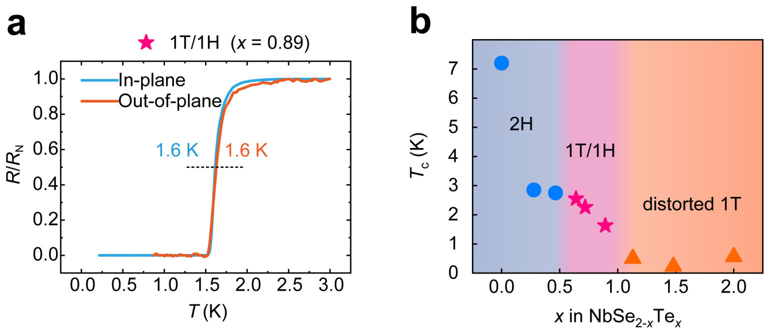

Supplementary Fig. 17 | Superconductivity of $NbSe_{2-x}Te_x$ single crystals. a Normalized resistance (R_N representing the normal-state resistance) as a function of temperature for both in-plane (blue line) and out-of-plane (red line) measurements. These results indicate a superconducting transition at 1.6 K in the 1T/1H superlattice ($x = 0.89$). The superconducting transition temperatures of the 1T/1H superlattice are nearly identical along different crystal orientations. **b** Structural phase and superconducting transition temperature as functions of doping level x in $NbSe_{2-x}Te_x$.

Supplementary References

1. Wilson, J. A. & Yoffe, A. D. The transition metal dichalcogenides discussion and interpretation of the observed optical, electrical and structural properties. *Adv. Phys.* **18**, 193-335 (1969).
2. Katzke, H., Toledano, P. & Depmeier, W. Phase transitions between polytypes and intralayer superstructures in transition metal dichalcogenides. *Phys. Rev. B* **69**, 13 (2004).
3. Cukjati, D. et al. The instability of the $NbTe_2$ surface structure. *Phys. Status Solidi* **193**, 246-250 (2002).
4. Nakata, Y. et al. Monolayer 1T-$NbSe_2$ as a Mott insulator. *NPG Asia Mater.* **8**, e321 (2016).
5. Ekvall, I., Kim, J.-J. & Olin, H. a. Atomic and electronic structures of the two different layers in $4H_b$-TaS_2 at 4.2 K. *Phys. Rev. B* **55**, 6758-6761 (1997).
6. Nayak, A. K. et al. Evidence of topological boundary modes with topological nodal-point superconductivity. *Nat. Phys.* **17**, 1413-1419 (2021).
7. Yan, D. et al. NbSeTe—a new layered transition metal dichalcogenide superconductor. *J. Phys.: Condens. Matter* **32**, 025702 (2020).
